# Galectin-1 is expressed in early-type neural progenitor cells and down-regulates neurogenesis in the adult hippocampus

**DOI:** 10.1186/1756-6606-4-7

**Published:** 2011-01-27

**Authors:** Yoichi Imaizumi, Masanori Sakaguchi, Tsuyoshi Morishita, Mamoru Ito, Françoise Poirier, Kazunobu Sawamoto, Hideyuki Okano

**Affiliations:** 1 Department of Physiology, Keio University School of Medicine, Tokyo, Japan; 2 Bridgestone Laboratory of Developmental and Regenerative Neurobiology, Keio University School of Medicine, Tokyo, Japan; 3 Development Division, Kyowa Hakko Kirin Co., Ltd., Tokyo, Japan; 4 Central Institute for Experimental Animals, Kanagawa, Japan; 5 Institut Jacques Monod, UMR CNRS 7592, University Paris Diderot, Paris, France; 6 Department of Developmental and Regenerative Biology, Nagoya City University Graduate School of Medical Sciences, Aichi, Japan

## Abstract

**Background:**

In the adult mammalian brain, neural stem cells (NSCs) proliferate in the dentate gyrus (DG) of the hippocampus and generate new neurons throughout life. A multimodal protein, Galectin-1, is expressed in neural progenitor cells (NPCs) and implicated in the proliferation of the NPCs in the DG. However, little is known about its detailed expression profile in the NPCs and functions in adult neurogenesis in the DG.

**Results:**

Our immunohistochemical and morphological analysis showed that Galectin-1 was expressed in the type 1 and 2a cells, which are putative NSCs, in the subgranular zone (SGZ) of the adult mouse DG. To study Galectin-1's function in adult hippocampal neurogenesis, we made *galectin-1 *knock-out mice on the C57BL6 background and characterized the effects on neurogenesis. In the SGZ of the *galectin-1 *knock-out mice, increased numbers of type 1 cells, DCX-positive immature progenitors, and NeuN-positive newborn neurons were observed. Using triple-labeling immunohistochemistry and morphological analyses, we found that the proliferation of the type-1 cells was increased in the SGZ of the *galectin-1 *knock-out mice, and we propose that this proliferation is the mechanism for the net increase in the adult neurogenesis in these knock-out mice DG.

**Conclusions:**

Galectin-1 is expressed in the neural stem cells and down-regulates neurogenesis in the adult hippocampus.

## Background

New neurons are continuously generated in the two neurogenic regions of the adult mammalian brain: the subgranular zone (SGZ) of the hippocampal dentate gyrus (DG) [1-3] and the subventricular zone (SVZ) of the lateral ventricle [4-7]. The neurons arising in these two systems have distinct roles in, respectively, odor discrimination or learning and memory [8-10]. Galectin-1 is expressed in the neural progenitor cells (NPCs) in both the SVZ of the lateral ventricle and the hippocampal SGZ [11,12]. Recent studies from our group have shown that Galectin-1 has therapeutic potential for treating neurodegenerative disorders (i.e., brain ischemia and spinal cord injury) via its ability to modulate neurogenesis [13,14]. Thus, studying the function of Galectin-1 in adult neurogenesis may contribute not only to elucidating the mechanism of adult neurogenesis but also to developing therapeutic strategies for brain repair [15,16].

Galectin-1 has distinct functions that depend on its redox state [17,18]. We reported that the reduced form of Galectin-1 promotes the proliferation of the NSCs in the SVZ through binding to the carbohydrate moieties of β1 Integrin [12,19]. This Galectin-1 function is crucial for functional recovery after brain ischemia [13]. On the other hand, the oxidized form of Galectin-1, which does not have the carbohydrate-binding activity, down-regulates cell proliferation through the cell-cycle arrest [20] of various cell types, including cerebellar astrocytes [21]. However, the detailed expression patterns and functions of Galectin-1 in adult hippocampal neurogenesis have been unclear.

Here, we studied the expression of Galectin-1 in the adult hippocampal SGZ and its role in adult hippocampal neurogenesis. We used multi-color immunohistochemistry to study neurogenesis in *galectin-1 *knock-out mice on the C57BL/6 background, and found that Galectin-1 is expressed in early-type NPCs of the adult mouse hippocampus and down-regulates neurogenesis.

## Results

### Galectin-1 was expressed in type 1 and 2a cells in the DG of the adult mouse brain

Previous studies showed that Galectin-1 is expressed in the hippocampal NPCs; however, the exact cell type(s) that express Galectin-1 has not been clear [11,12]. Since hippocampal neurogenesis has been well characterized in C57BL/6 mice [22,23], we produced *galectin-1 *knock-out mice on the C57BL/6 background using a speed back-cross technique [24] combined with selection for the enriched transmission of 64 microsatellite markers that distinguish C57BL/6 from the 129 background (Materials and Methods, See Additional file 1, Table 1).

**Figure 1:**
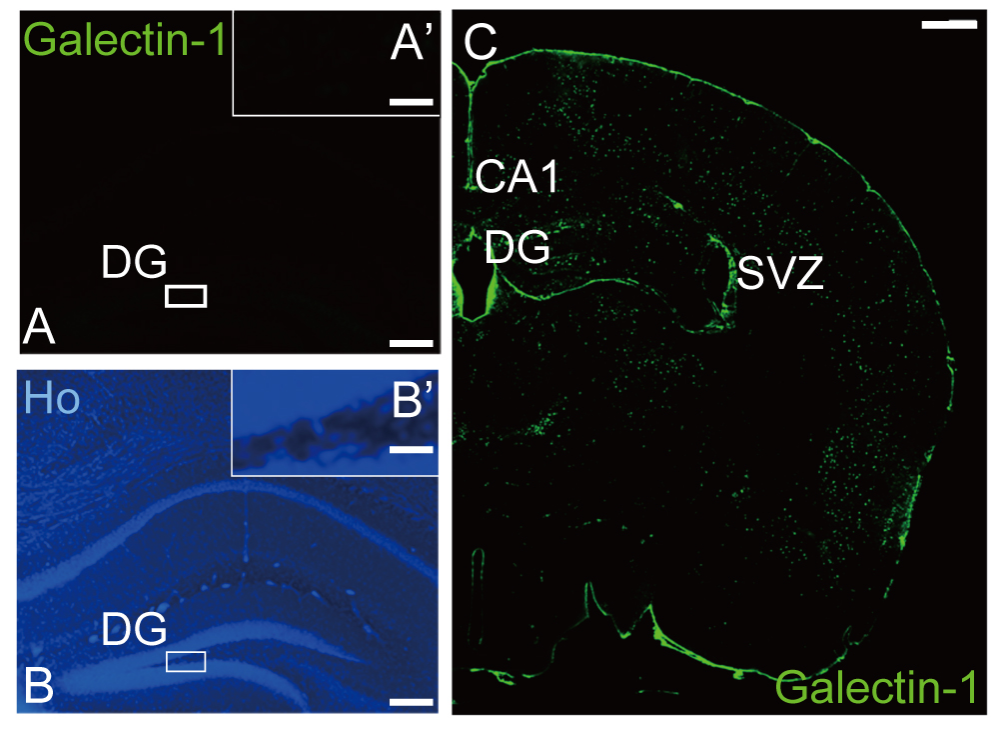
**Galectin-1 is expressed in the adult hippocampus**. (A, B) Galectin-1 (A) and nuclear (B) staining in the hippocampus of the galectin-1-null mouse DG. (A', B') High-magnification images of the boxed regions, respectively. No Galectin-1+ cells were detected in the DG of galectin-1 null mutants, indicating that the staining was specific for Galectin-1. (C) Low-magnification images of Galectin-1+ cells in a coronal section of the adult mouse brain. Galectin-1 was expressed in the cortex, SVZ, and hippocampus. (Scale bars: A, B, 500 μm; A', B', 50 μm; C, 1000 μm).

**Figure 2:**
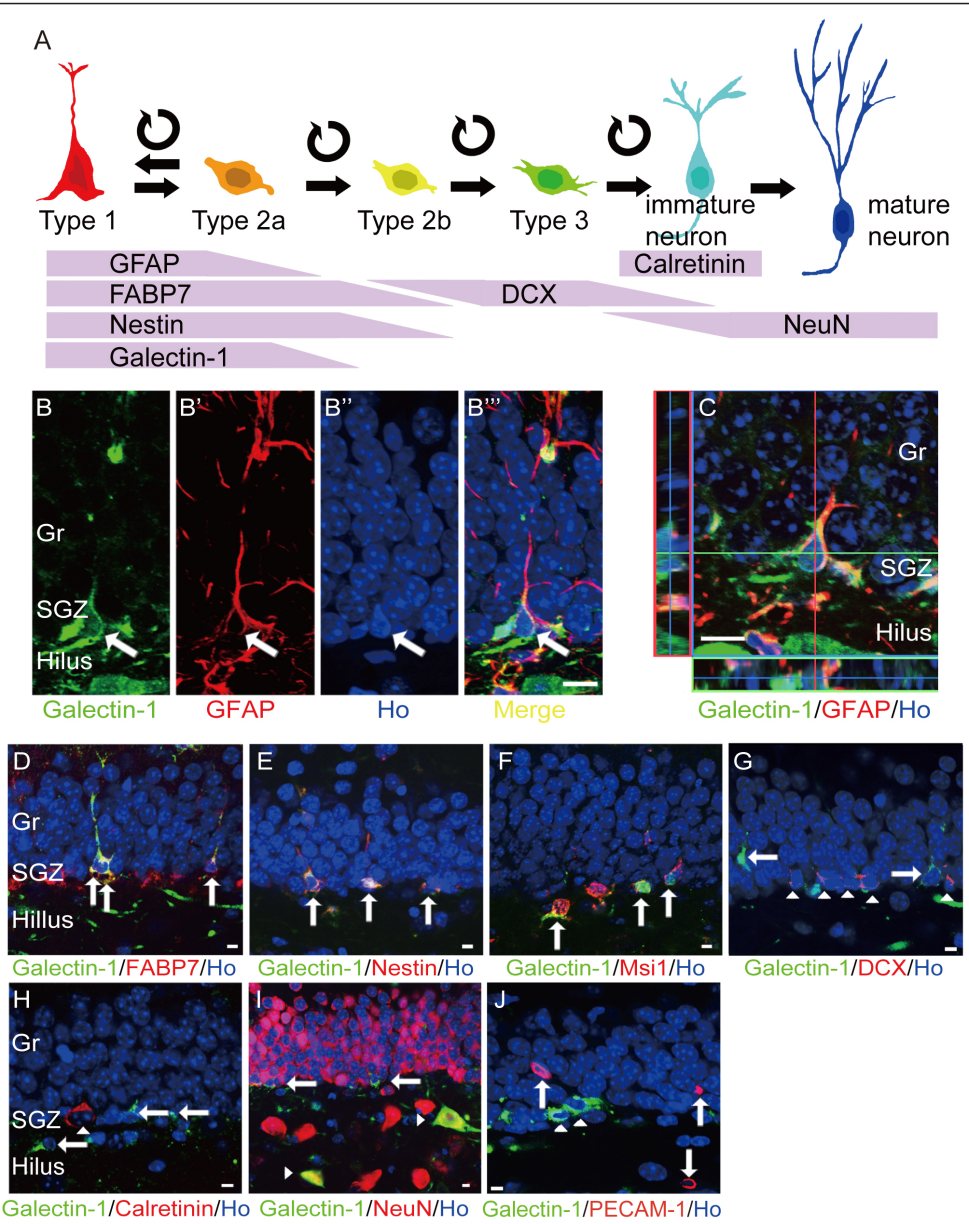
**Galectin-1 is expressed in type 1 and 2a cells in the adult hippocampus**. (A) Schematic diagram of neural marker expression in the adult DG, correlated with neuronal cell stage. DG, dentate gyrus; LV, lateral ventricle; Gr, granule layer; SGZ, subgranular zone (B-C) Three-dimensional reconstructions from confocal optical images of Galectin-1 (green) and GFAP (red) double-labeling, and Hoechst (blue) nuclear staining in the hippocampus. (B) Galectin-1+/GFAP+ cells were seen in the DG (arrow). (C) A projected image from one optical slice of B. (D-J) Galectin-1 expressed in type 1 and 2a cells in the adult DG. Arrows indicate FABP7 (red) and Galectin-1 (green) double-positive (yellow) cells (D), Nestin (red) and Galectin-1 (green) double-positive (yellow) cells (E), and Msi1 (red) and Galectin-1 (green) double-positive (yellow) cells (F) in the DG. (G) No co-labeling for Galectin-1+ (green; arrows) and DCX+ (red; arrowheads) in DG cells was seen. (H) Galectin-1+ (green; arrows) cells did not express Calretinin (red; arrowhead). (I) Galectin-1 (green; arrows) was not expressed in NeuN+ (red) cells. Note the presence of Galectin-1-positive interneurons in the hilus (arrowheads). (J) Galectin-1+ (green; arrowheads) cells did not express the blood vessel marker PE-CAM1 (red; arrows). DG, dentate gyrus; LV, lateral ventricle; Gr, granule layer; SGZ, subgranular zone. (Scale bars: B-C, 10 μm; D-J, 5 μm).

A Galectin-1-specific antibody did not label tissue in the *galectin-1 *knock-out mouse brain (Fig. 1 A-1B'), indicating that our staining procedure was able to specifically label Galectin-1 but not other proteins of Galectin family. As we reported previously [12], Galectin-1+ cells were found in the cortex, SVZ, and DG of the wild type control mouse brain (Fig. 1 C). To study which cell type(s) in the DG expressed Galectin-1, we performed double-immunostaining for Galectin-1 and several cell-type-specific markers (Fig. 2 A), including GFAP, which is expressed in the type 1 and 2a cells, Nestin and FABP7, which are expressed in the type 1, 2a, and 2b cells [25], and Musashi1 (Msi1), which is expressed in early-stage NPCs [26,27]. Subsets of Galectin-1+ cells were positive for GFAP (Fig. 2 B-B''', C), FABP7, Nestin, and Msi1 (Fig. 2 D-F). Cells expressing DCX, which are type 2b and 3 cells and immature neurons [25,28], and Calretinin, which are post-mitotic immature neurons [29], were never co-labeled with Galectin-1 (Fig. 2 G, H). Although some NeuN+ interneurons in the hilus were Galectin-1+, the NeuN+ cells in the SGZ and the granular cell layer were not co-labeled with Galectin-1 (Fig. 2 I). PE-CAM1+ cells, which are endothelial cells of blood vessels [30], did not co-label with Galectin-1 in the DG (Fig. 2 J). Taken together, these results suggest that, in the DG, type 1 and/or 2a cells express Galectin-1.

Next, we examined the expression of Galectin-1 in the type 1 and 2a cells more closely. Since the identification criteria for type 1 cells depends on their morphology (a triangular cell soma and an apical process) [25], and the Galectin-1 immunoreactivity does not clearly stain cell processes, we performed double-labeling with Galectin-1 and FABP7, GFAP, or Nestin, which are commonly used for the morphological analysis of hippocampal progenitors, to identify the type 1 cells [25] (also see Materials and Methods). We found that the anti-FABP7 antibody labeled both the cell soma and the processes of Galectin-1+ cells more clearly than the anti-GFAP or anti-Nestin antibody (Fig. 2 B''', D, E); therefore, we used the anti-FABP7 antibody in the subsequent analyses.

Among the Galectin-1+ cells in the DG, 7.3% ± 1.4% (*n *= 5 mice) had the characteristic triangular soma and apical process of type 1 cells (Fig. 2 D, Materials and Methods) [31]. The majority of the other Galectin-1+ cells had a non-radial cell shape, a small cell soma, and an irregularly shaped nucleus, which are characteristics of type 2 cells (Fig. 2 E, F) [31,32]. In the DG, of the cells that showed the typical type-1 cell morphology, 33.1% ± 5.6% (*n *= 5 mice) expressed Galectin-1, indicating that a subset of the type 1 cells expresses Galectin-1.

### Adult neurogenesis increased in the SGZ of the *galectin-1*-null mutant mice

The expression pattern of Galectin-1 in the DG led us to examine its function in the NPCs in adult hippocampal neurogenesis. BrdU, which is incorporated by proliferating cells, can be used to quantify adult neurogenesis in the DG [28]. Therefore, we performed a series of BrdU pulse-chase experiments [28] using the *galectin-1*-null mutant mice and their control littermates. To study each stage of NPC maturation separately, the mice were sacrificed at two different time points after BrdU infusion, and multi-color immunohistochemistry was performed to visualize the incorporated BrdU in the context of stage-specific marker expression (i.e., FABP7, DCX, or NeuN) (Fig. 2 A).

**Figure 3:**
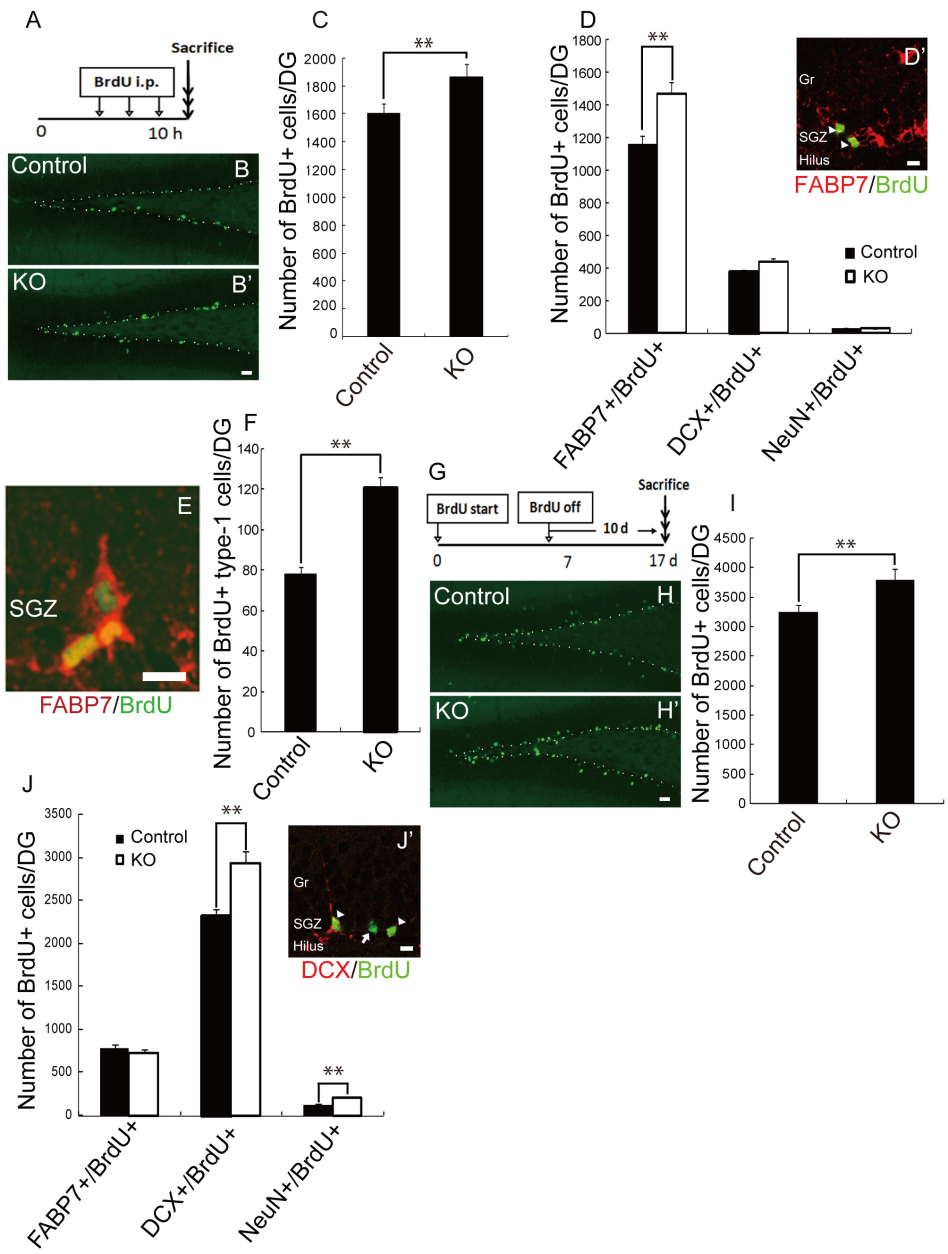
**The number of NPCs in the DG was higher in the *galectin-1 *null mutant mouse than in controls**. (A-D') The increase in the number of FABP7+/BrdU+ cells in the *galectin-1 *null mutant mouse brain was significant. (A) Time-line for the BrdU infusion experiment. BrdU was infused every 3.3 hours for 10 hours, and the mice were sacrificed 3.4 hours after the last injection. (B, B') BrdU+ cells in the DG of a *galectin-1*-null mutant mouse (B') and control (heterozygous) mouse (B). n = 5 mice each. Dotted lines outline the SGZ. (C) The number of BrdU+ cells in the DG of the KO mice was greater than in their heterozygous littermates. *P *< 0.05; *n *= 5 mice each. (D) The number of FABP7+/BrdU+ cells was greater in the DG of *galectin-1 *null mutant mice than in that of controls. *P *< 0.05; *n *= 5 mice each. (D') Double-labeling for FABP7 (red) and BrdU (green). Arrowheads indicate double-labeled cells. (E) High-power image of FABP7+/BrdU+ double-labeled cells in the SGZ. (F) The number of BrdU+/type 1 cells was greater in the *galectin-1*-null mutant mouse DG than in that of controls. *P *< 0.05; *n *= 5 mice each. (G) Time-line for the long-term BrdU infusion experiment. BrdU was given for 7 days, and the mice were sacrificed 10 days after the last day of BrdU administration. (H, H') BrdU+ cells in the galectin-1 null mutant mouse DG (H') and control mouse DG (H). *P *< 0.05; *n *= 5 mice each. Dotted lines outline the SGZ. (I) The number of DCX+/BrdU+ cells was greater in the *galectin-1*-null mouse DG. *P *< 0.05; *n *= 5 mice each. (J) Quantitative comparison of FABP7+/BrdU+, DCX+/BrdU+, and NeuN+/BrdU+ cells in the DG of the KO and control mice. Significant increases were seen in the DCX+/BrdU+ and NeuN+/BrdU+ cells in the KO DG. (J') Arrowheads indicate DCX (red) and BrdU (green) double-positive cells, and the arrow indicates a BrdU single-positive cell. (Scale bars: B, B', H, H', 100 μm; D', E, J', 5 μm).

In the first BrdU-pulse chase experiment, BrdU was injected every 3.3 hours for 10 hours, and the mice were sacrificed 3.4 hours after the last injection (Fig. 3 A) [12,33]. As previously described [28,34], in the control DG, the BrdU+ cells were mainly early-stage NPCs (Fig. 3 D'). Interestingly, the total number of BrdU+ cells in the SGZ was higher in the *galectin-1*-null mutant mice (Fig. 3 B-C; P < 0.05; n = 5 mice each), as was the number of FABP7+/BrdU+ double-positive cells (Fig. 3 D, D'; P < 0.05; n = 5 mice each). By morphological analysis of these FABP7+/BrdU+ cells ([25]; Materials and Methods), we found that the number of BrdU+ type 1 cells also increased in the *galectin-1*-null mutants (Fig. 3 E, F; P < 0.01; n = 5 mice each), suggesting that NSCs also increased in the null mutant mouse DG.

In the second BrdU-pulse chase experiment, BrdU was given in the drinking water for 7 days, and the mice were sacrificed 10 days after the last day of BrdU administration (Fig. 3 G) [28,34]. As expected, in the wild-type DG, the BrdU+ cells were mostly late-stage NPCs (Fig. 3 J'). The total number of BrdU+ cells in the SGZ increased in the *galectin-1 *null mutants (Fig. 3 H-I; P < 0.05; n > 5 mice each) as did the number of DCX+/BrdU+ cells and NeuN+/BrdU+ cells (Fig. 3 J, J'; P < 0.05; n > 5 mice each). Taken together, these results suggested that in the absence of Galectin-1, hippocampal neurogenesis is increased.

### Proliferation of type 1 and 2a cells was up-regulated in the *galectin-1*-null mutant mice

**Figure 4:**
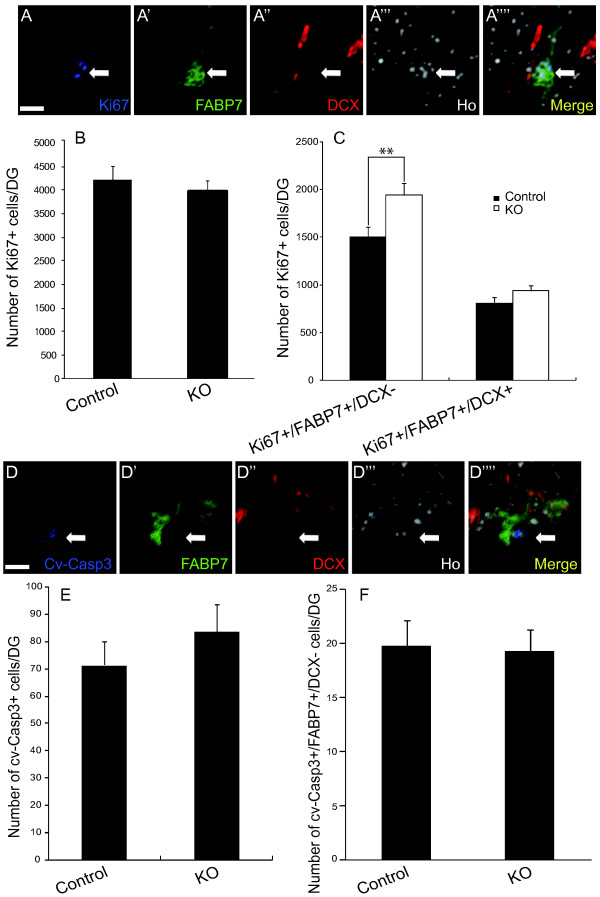
**The number of proliferating type 1 and 2a cells was greater in the *galectin-1*-null mutant DG than the control DG**. (A-A'''') High-magnification images of Ki67 (blue), FABP7 (green), and DCX (red) triple staining with nuclear Hoechst (white) in the DG. Arrow indicates a Ki67+/FABP7+/DCX- cell. (B) The total number of Ki67+ cells was not different between the *galectin-1 *heterozygous and *galectin-1*-null mice. *P *= 0.25; *n *> 5 mice each. (C) The number of Ki67+/FABP7+/DCX- cells was higher in the *galectin-1 *null mutants, suggesting that Galectin-1 negatively regulates the proliferation of type 1 and 2a cells in the adult DG. *P *< 0.05; *n *> 5 mice each. *P *< 0.01; *n *> 5 mice each. (D-D'''') High-magnification images of cv-Casp3 (blue), FABP7 (green), and DCX (red) triple staining, with nuclear Hoechst (white) in the DG. Arrow indicates a cv-Casp3+/FABP7+/DCX- cell. (E) The number of cv-Casp3+ cells was comparable between the *galectin-1 *heterozygous and KO mice. *P *= 0.12; *n *> 11 mice each. (G) No significant difference between the number of cv-Casp3+/FABP7+/DCX- cells in the SGZ of *galectin-1 *KO and control mice. *n *> 22 mice each. (Scale bars: A-A'''', D-F'''', 5 μm)

To analyze the mechanism of the increased adult hippocampal neurogenesis in the *galectin-1 *null mutant mice, we studied the proliferation status of NPCs in the SGZ by multi-color immunohistochemistry. The total number of Ki67+ cells was comparable between the *galectin-1*-null mutants and their control littermates (Fig. 4 A-B; P = 0.25; n > 5 mice each). However, triple-labeling with Ki67, FABP7, and DCX revealed that the number of proliferating FAPB7+/DCX- cells, which represent cell types 1 and 2a (Fig. 4 A-A'''') [25], increased in the *galectin-1*-null mutants (Fig. 4 A-A'''', C; P < 0.05; n > 5 mice each). On the other hand, the number of proliferating FABP7+/DCX+ cells, which represent the type 2b cells, was comparable between the *galectin-1*-null mutants and their control littermates (Fig. 4 C; P = 0.08; n > 5 mice each) [25]. These results suggest that the absence of Galectin-1 resulted in an increase of type 1 and/or 2a cell proliferation, but not of type 2b cell proliferation in the DG.

To examine apoptosis in the adult neurogenic regions of the *galectin-1 *null mutants, we performed immunostaining for cleaved-Caspase-3 (cv-Casp3), a marker for apoptotic cells [35]. The total number of cv-Casp3+ cells in the SGZ was comparable between the *galectin-1*-null mutants and their control littermates (Fig. 4 D-E; P = 0.18; n > 22 mice each). In addition, the number of FABP7/DCX/cv-Casp3 triple-positive cells, which represent apoptotic type 1 and 2a cells, was also comparable (Fig. 4 D-D'''', F; P = 0.44; n > 22 mice each).

Collectively, these data suggest that the absence of Galectin-1 resulted in an increase in the proliferation of the type 1 and/or 2a cells in the DG of the *galectin-1 *null mutant mice.

## Discussion

**Figure 5:**
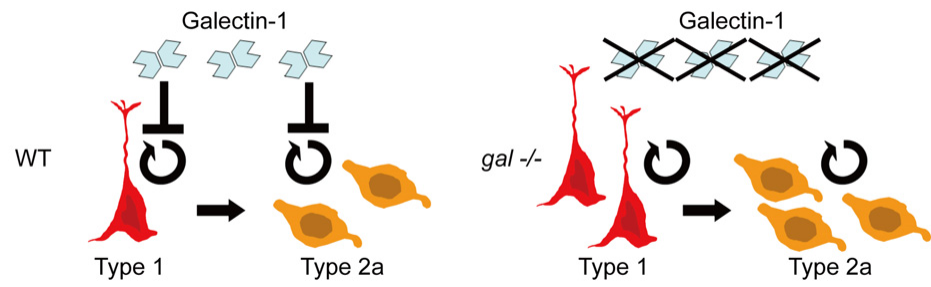
**Schematic diagram showing the expression and function of Galectin-1 in adult neurogenesis in the DG**. Galectin-1 is expressed in type 1 and 2a cells and inhibits their proliferation under normal conditions (left). In the *galectin-1*-null mutant DG, the absence of Galectin-1 leads to an increased number of neural precursor cells (right).

In this study, we showed that Galectin-1 is expressed in early-type of NPCs (i.e., type 1 and 2a cells) in the adult mouse hippocampus. By using *galectin-1 *knock-out mice on the C57BL/6 background, we showed that Galectin-1 is required for the normal proliferation of the early NPCs of the adult hippocampus. These results suggest that Galectin-1 suppresses the proliferation of early-type of the NPCs during adult hippocampal neurogenesis (Fig. 5).

Recent studies reported that type 1 and 2a cells act as stem cells in the adult DG [25,36-38]. Our immunohistochemical and morphological studies showed that Galectin-1 is expressed by a subset of type1 and 2a cells in the DG, suggesting that Galectin-1 is expressed in NSCs. We found that the increased proliferation in the DG of *galectin-1 *null mutants led to larger numbers of type 1 cells and their daughters, including newborn neurons. Previous reports showed that Galectin-1 can regulate the cell-cycle and/or apoptosis, depending on the biological context [20,39-41]. We found that although the proliferation of the type 1 and 2a cells increased, apoptosis of these cells remained normal in the *galaectin-1 *knock-out mouse DG. These results suggest that the increased numbers of type 1 and 2a cells, and their progenies in the DG of the knockout mice was caused by the increased proliferation of the type1 and 2a cells.

Surprisingly, our study showed an opposite function of Galectin-1 in the adult DG compared with reports on Galectin-1's function in the adult SVZ [12,13]. Previously, Galectin-1 was reported to have biphasic functions in cell growth, depending on its redox state [41]. At low concentrations (~1 ng/ml), Galectin-1 exists in its reduced form and possesses a mitogenic activity; at high concentrations (~10 μg/ml), Galecitn-1 is converted to an oxidized form and arrests the cell-cycle [20,42]. Consistent with these findings, relatively high concentrations of Galectin-1 (1 and 10 μg/ml) decreased the number of adult hippocampal NPCs *in vitro *(data not shown). In contrast, low-concentration Galectin-1 (10 and 100 ng/ml) increases the number of SVZ NPCs *in vitro *[12,19], and infusion of a reduced form of Galectin-1 (CS-Galectin-1) promotes the proliferation of NSCs in the adult SVZ *in vivo *[12]. These findings suggest that the reduced form of Galectin-1 promotes the proliferation of NPCs in the adult SVZ, whereas the oxidized form of Galectin-1 inhibits the proliferation of adult hippocampal NPCs. The validity of this hypothesis will be clarified in a future study.

A recent report showed a decrease of BrdU-positive NPCs in the DG of *galectin-1 *knockout mice on a 129 background [11]. Although 129 mouse lines are widely used in biology, they are known to show poor hippocampus-dependent learning [43,44], low levels of activity [45], and altered long-term potentiation in hippocampal neurons [46]. On the other hand, the adult hippocampal neurogenesis in the C57BL/6 mouse is well characterized [22,23]. The C57BL/6 line not only has a high rate of the NPC proliferation in the SGZ but also displays experience-related regulation of cell survival and a comparatively high rate of net neurogenesis in the adult DG [22,23]. To avoid confounding data from background-dependent phenotypes, we generated the *galectin-1 *knock-out mouse line on the C57BL/6 background for our analysis of the expression and function of Galectin-1 in adult hippocampal neurogenesis. This led to our identification of a new function of Galectin-1 in adult hippocampal neurogenesis.

There are some molecules that function in both the SVZ and DG in adult neurogenesis (TGFβ, Notch [47,48]); however, no molecule has been described to date that functions in opposite directions in the two neurogenic systems (i.e., up-regulates neurogenesis in the SVZ and down-regulates it in the SGZ, as in Galecitn-1's case). Our findings may help to elucidate differences in the mechanisms of neurogenesis in these two major neurogenic regions of the adult brain [15,16].

## Conclusions

Our study demonstrated that Galectin-1 was expressed in the early-type NPCs (i.e. type 1 and 2a cells) in the adult DG. Moreover, we showed that the number of type 1 and 2a cells increased in the adult DG of the *galectin-1 *knockout mice. These results suggest that Galectin-1 negatively regulates the proliferation of the early-type NPCs in adult hippocampal neurogenesis. These findings represent a new step toward understanding the mechanism of adult neurogenesis and toward establishing new methods to repair the adult CNS after injury and to treat degenerative neurological disorders using endogenous molecules.

## Materials and methods

### Immunohistochemistry

Brains were perfusion-fixed with 4% paraformaldehyde (PFA), postfixed in the same fixative overnight, and then cut into 50-μm sections on a vibratome. After three rinses in PBS, the sections were incubated for 20 min in TNB blocking solution (Vector Laboratories), incubated with primary antibodies overnight, and then incubated for 60 min at room temperature with the Fab2-portion of secondary antibodies (1:500; Jackson ImmunoResearch) conjugated with HRP (1:500; Jackson ImmunoResearch) or Alexa Fluor (1:200; Molecular Probes), unless otherwise noted. The biotin- and HRP-conjugated antibodies were visualized using the Vectastain Elite ABC kit and/or TSA (Vector Laboratories). For multi-color labeling, the potential for the cross-reactivity of the secondary antibodies with off-target primary antibodies was carefully tested and excluded by using the appropriate controls (e.g., parallel staining without one of the primary antibodies).

### **Primary Antibodies**

The primary antibodies (final dilution and source) used in this study were as follows: mouse monoclonal anti-GFAP (1:200, Sigma); rat monoclonal anti-Bromodeoxyuridine (BrdU) (1:250, Abcam); rabbit polyclonal anti-Calretinin (1:100, Swant); goat polyclonal anti-DCX (1:300, Santa Cruz Biotechnology); rabbit polyclonal anti-PE-CAM-1 (1:100, Beckton Dickinson); rat monoclonal anti-Musashi1 (1:500, [26,49,50]); rabbit polyclonal anti-FABP7 (1:200, Chemicon); rabbit polyclonal anti-Ki67 (1:5000, Abcam); goat anti-Galectin-1 (1:200, R&D Systems); mouse monoclonal anti-Nestin (1:100, Developmental Studies Hybridoma Bank); mouse monoclonal anti-NeuN (1:100, Chemicon); and rabbit polyclonal anti-cleaved-Caspase-3 (1:200, Cell Signaling Technology).

### BrdU pulse-chase labeling

For the short-term chase, mice were given i.p. injections of BrdU (120 mg/kg dissolved in phosphate buffer; Sigma) every 3.3 h for 10 h and were sacrificed 3.4 h after the last injection. For the intermediate-term chase, 1 mg/ml BrdU was given to mice in their drinking water for 1 or 2 weeks. The mice were killed 10 days after the last day of BrdU administration, and the brains were processed for immunohistochemistry.

### Quantification of histological results

To quantify each cell type, 40 coronal vibratome sections of the DG (50-μm thick) were obtained at the level of the caudate-putamen (1.2 to 3.2 mm caudal to the bregma) from each hemisphere. In the BrdU experiments, the sections were double labeled for FABP7/BrdU, DCX/BrdU, or NeuN/BrdU. First, we counted the total BrdU-positive nuclei (Apotome, Zeiss) in each section. Next, several images were captured as 1.5-μm optical sections (LSM-510, Zeiss), and the BrdU+ nuclei that were positive for each of the markers (FABP7, DCX, NeuN) were counted. The total number of BrdU+ cells was then multiplied by the ratio of the cells of each type to BrdU+ cells, yielding the numbers for each cell type, as follows: Type 1, 2a, 2b cells = total number of BrdU+ (FABP7+/BrdU+); Type 2b, 3 cells and immature neurons = total number of BrdU+ (DCX+/BrdU+); and mature neurons = total number of BrdU+ (NeuN+/BrdU+). For each experiment, the numbers of BrdU+ cells were normalized to the total number of BrdU+ cells in a heterozygous mouse in a single 50-μm slice.

Proliferating cells were detected using an anti-Ki67 antibody. The sections were triple stained for FABP7, DCX, and Ki67. We counted the total number of Ki67+ nuclei (Apotome, Zeiss) in each vibratome section. Several images were captured as 1.5-μm optical sections (LSM-510, Zeiss), and the number of Ki67+ nuclei that were positive for each of the markers (FABP7, DCX) was counted. The total number of Ki67+ cells was multiplied by the ratio of the cells of each type to Ki67+ cells, yielding the number of each cell type, as follows: type 1, 2a cells = total number of Ki67+ (FABP7+/DCX-/Ki67+); type 2b cells = total number of Ki67+ (FABP7+/DCX+/Ki67+).

Apoptotic cells were detected using an anti-cv-Casp-3 antibody. The sections were triple stained for cleaved Casp-3, FABP7, and DCX. We counted the total cv-Casp-3-positive nuclei and the number of cv-Casp-3+ nuclei that were positive for each marker (FABP7, DCX), as described above. The total number of cv-Casp-3+ cells was multiplied by the ratio of the number of cells of each type to cv-Casp-3+ cells, yielding the number of each cell type, as follows: Type 1, 2a cells = total number of cv-Casp-3+ (FABP7+/DCX-/cv-Casp-3+).

To quantify the type 1 cells, we counted the number of FABP7+/Galectin-1+ (/Ki67+) or FABP7+/BrdU+ radial glia-like cells, which have a triangular cell soma and apical process, in the SGZ [25]. By following our criteria for the type 1 cells, we found that only 7.1% ± 2.9% (n = 5 mice) of the total type 1 cell population was proliferating in control mice. This data are consistent with a previous report [28].

### Generation of galectin-1 knock-out mice in the C57BL6/J background

To generate *galectin-1-*null homozygous C57BL6/J mice from 129SJ mice [51], we used the speed back-cross technique [24] and monitored 64 microsatellite markers from the Mouse Genome Informatics database (http://www.informatics.jax.org/) that can be used to discern the 129SJ and C57BL/6J strains (See Additional file 1, Table 1). The markers were analyzed using the ABI 3100 Genetic Analyzer, ABI GeneScan 3.7, and Genotyper 2.5 software. After the fifth backcrossing with selected mice that showed the best B6-exchanged PCR results in each backcross step, we obtained mice in which all 64 129SJ markers had been replaced with the C57BL/6J markers.

### Animals

For the adult mouse study, 8-week-old male mice were killed by anesthetic overdose. We used heterozygous mice as controls. Mice were maintained on a 12-h light/12-h dark cycle with unlimited access to food and water. All the experiments on live animals were performed in accordance with Keio University guidelines and regulations.

### Statistical analysis

Values are expressed as the mean ± SE. An unpaired t-test (for two groups) or ANOVA with the Bonferroni correction (for more than three groups) was used to evaluate the differences between averages unless otherwise noted.

## Competing interests

The authors declare that they have no competing interests.

## Authors' contributions

Author contributions: YI, MS, and HO, designed the research. YI, MS, and TM performed the research; YI analyzed all the data; MI and FP, prepared the *galectin-1 *knockout mouse, YI, MS, KS, and HO prepared the manuscript. All authors read and approved the final manuscript

## Supplementary Material

Additional file 1 **Table 1**. Click here for file
