# Supplementary material for: Galectin-1 is expressed in early-type neural progenitor cells and down-regulates neurogenesis in the adult hippocampus
Source: Mol Brain. 2011 Jan 27;4:7. doi: 10.1186/1756-6606-4-7 (PMC3041742; doi:10.1186/1756-6606-4-7)
Supplement: Additional file 1 — Table 1. [file 1756-6606-4-7-S1.DOC]

| List of microsatellite markers |
| --- |
| D1Mit67, D1Mit303, D1Mit91, D1Mit459 (chromosome 1), D2Mit312, D2Mit182, D2Mit311, D2Mit346 (chromosome 2), D3Mit62, D3Mit25, D3Mit85, D3Mit89 (chromosome 3), D4Mit227, D4Mit52, D4Mit190 (chromosome 4), D5Mit1, D5Mit58, D5Mit367, D5Mit97 (chromosome 5), D6Mit86, D6Mit284, D6Mit304 (chromosome 6), D7Mit193, D7Mit100, D7Mit189 (chromosome 7), D8Mit217, D8Mit191, D8Mit93 (chromosome 8), D9Mit250, D9Mit8, D9Mit215 (chromosome 9), D10Mit2, D10Mit31, D10Mit266 (chromosome 10), D11Mit21, D11Mit67, D11Mit184 (chromosome 11), D12Mit109, D12Mit156, D12Mit30 (chromosome 12), D13Mit132, D13Mit13, D13Mit51 (chromosome 13), D14Mit1, D14Mit233, D14Mit225 (chromosome 14), D15Mit12, D15Mit85, D15Mit171 (chromosome 15), D16Mit129, D16Mit48, D16Mit106 (chromosome 16), D17Mit163, D17Mit138, D17Mit53 (chromosome 17), D18Mit19, D18Mit40, D18Mit25 (chromosome 18), D19Mit78, D19Mit14, D19Mit103 (chromosome 19), DXMit55, DXMit25, DXMit130 (chromosome X) |
